# Characterization of new mouse models of acute and chronic *Mycobacterium abscessus* infection for antimicrobial drug screening

**DOI:** 10.1128/aac.00475-25

**Published:** 2025-09-26

**Authors:** Ilham M. Alshiraihi, Ha Lam, Brennen T. Troyer, Kristina N. Tran, Malik Zohaib Ali, Daryl K. Conner, Abigail Godelfer, Camron Pearce, Mary Jackson, Marcela Henao-Tamayo, Sara E. Maloney Norcross, Bernd Meibohm, Richard E. Lee, Mercedes Gonzalez-Juarrero, Andres Obregon-Henao

**Affiliations:** 1 Mycobacteria Research Laboratories, Non-Tuberculous Mycobacteria Center, Colorado State University, Fort Collins, Colorado, USA; 2 Microbiology, Immunology and Pathology, Colorado State University 3447, Fort Collins, Colorado, USA; 3 Technology Advancement and Commercialization, RTI International, Research Triangle Park, North Carolina, USA; 4 Department of Pharmaceutical Sciences, University of Tennessee Health Science Center 427811, Memphis, Tennessee, USA; 5 Department of Chemical Biology and Therapeutics, St. Jude Children’s Research Hospital 541707, Memphis, Tennessee, USA; Bill & Melinda Gates Medical Research Institute, Cambridge, Massachusetts, USA

**Keywords:** drug testing, preclinical, mouse, models, *Mycobacterium*, *abscessus*

## Abstract

*Mycobacterium abscessus* (MAB), a rapidly growing non-tuberculous mycobacterium, is becoming increasingly recognized as a significant pathogen affecting humans. These bacteria particularly impact individuals with cystic fibrosis (CF), non-CF bronchiectasis, and compromised immune systems. Treating pulmonary infections with MAB is challenging due to the bacteria’s inherent and acquired resistance to many antibiotics, including most anti-tuberculosis antibiotics. Antibiotic therapy of MAB infection is lengthy, involves multiple oral and parenteral administered drugs, induces significant toxicity, and, on many occasions, fails to cure. Consequently, developing more effective antibiotics has become a high priority. Preclinical studies to evaluate antibiotic efficacy against MAB are challenging because they fail to establish a progressive and sustained pulmonary infection in commonly used animal models. To address this issue, the course of MAB pulmonary infection was evaluated in 15 immunocompetent or deficient mouse strains. We report bacterial burden and histopathology and classify the models according to their ability to clear or sustain progressive infection beyond 28 days. We also examined the potential of these models for drug screening. Our findings provide a foundation for selecting suitable mouse models of pulmonary MAB infection for drug discovery.

## INTRODUCTION

In most instances, exposure to *Mycobacterium abscessus* (MAB) is controlled by the host, without causing progressive or sustained infection. However, MAB can establish infection in soft tissues and cause chronic pulmonary infections in individuals undergoing immunosuppressive chemotherapy or those with underlying structural lung abnormalities caused by bronchiectasis, chronic obstructive pulmonary disease, cystic fibrosis (CF), or prior tuberculosis (TB) disease (1). Current therapeutic options for patients with pulmonary MAB infection require prolonged administration of multiple oral and parenteral antibiotics over 12–24 months, often associated with poor bactericidal activity and significant toxicity. To date, treatment outcomes are generally poor with cure rates below 50% and with many patients relapsing after ending these very long and harsh treatments (2, 3).

New and effective multidrug therapies against MAB are urgently needed, but their development is heavily reliant on finding new or repurposed drugs that preferably do not require parenteral routes for administration. Rapid drug screening and testing, in turn, requires reliable *in vitro* and *in vivo* models. While murine models have been invaluable in TB drug discovery and effectively predicted the efficacy of new multidrug regimens in clinical trials (4–6), developing murine models of MAB is more challenging because this bacillus tends to be cleared in both immunocompetent and immunodeficient mouse strains.

The ideal animal model for drug testing should be highly reproducible, affordable, widely accessible to research laboratories, and rely on easy-to-procure animals in quantities necessary for most drug screening studies. Furthermore, most drugs are synthesized in limiting quantities for early stages of drug discovery; thus, smaller models are better to minimize the amount of drug required for *in vivo* testing. Because MAB can be found both intra- and extracellularly in patients, and drug efficacy varies depending on the cellular niche, the ideal lung infection model should harbor bacteria both within and outside cells. Finally, infection should induce progressive pulmonary pathology, enabling the evaluation of drug efficacy at different stages of infection and pathological conditions. Following these guidelines (7), we evaluated the course of pulmonary MAB infection in 15 mouse strains, including some with genetic mutations leading to immunodeficiency, lysosomal, and connective tissue disorders. We report the bacterial burden and histopathology and classified models based on their ability to sustain progressive infection or clear the bacteria within the first 28 days. Additionally, we examined the potential for monotherapy drug screening within the context of multidrug therapies commonly used in the clinics, including: the aminoglycoside amikacin (AMK), the macrolide clarithromycin (CLR), the tetracycline tigecycline (TGC), and the β-lactam imipenem (IMP). We also included the diarylquinoline bedaquiline (BDQ) because of its variable efficacy against MAB in clinical and preclinical studies (8, 9).

## RESULTS

**Fig 1:**
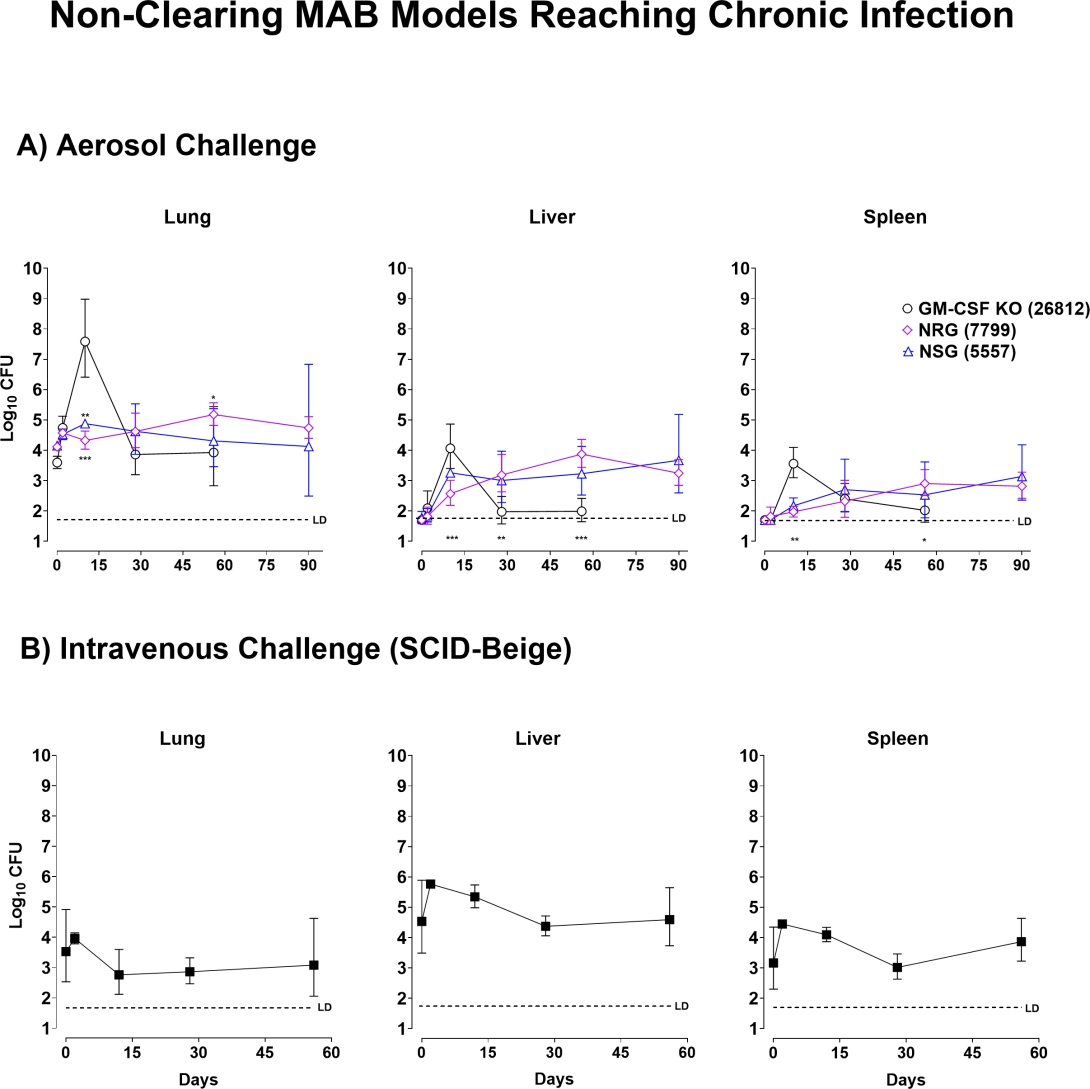
Murine models sustaining chronic (non-clearing) infection with *M. abscessus* ATCC 19977. (**A**) Bacterial load in granulocyte macrophage colony stimulating factor (GM-CSF) KO, NOD.Rag.IL2R gamma chain (NRG), and NOD.SCID.IL2R gamma chain (NSG) mice following aerosol challenge. Bacterial loads (*y*-axis: log_10_ CFU) in the lungs (left graph), livers (center graph), and spleens (right graph) of GM-CSF KO (black lines with circles), NRG (purple lines with rhomboids), and NSG (blue lines with triangles) mice (*n* = 5) following an aerosol challenge (~1 × 10^4^ CFU/lung) with *M. abscessus* ATCC 19977. Bacterial loads were measured at the indicated time points post-infection (*x*-axis; days). (**B**) Bacterial load in SCID-Beige mice following intravenous challenge. Bacterial loads (*y*-axis: log_10_ CFU) in the lungs (left graph), livers (center graph), and spleens (right graph) of SCID-Beige mice (*n* = 5) following intravenous exposure (~1 × 10^6^ CFU/mouse) with *M. abscessus* ATCC 19977. Bacterial loads were measured at days 0, 2, 12, and 28 post-infection (*x*-axis; days). The data are represented as mean log_10_ CFU with standard deviation. Differences between groups were analyzed by one-way analysis of variance with Tukey’s multiple comparison test.

**Fig 2:**
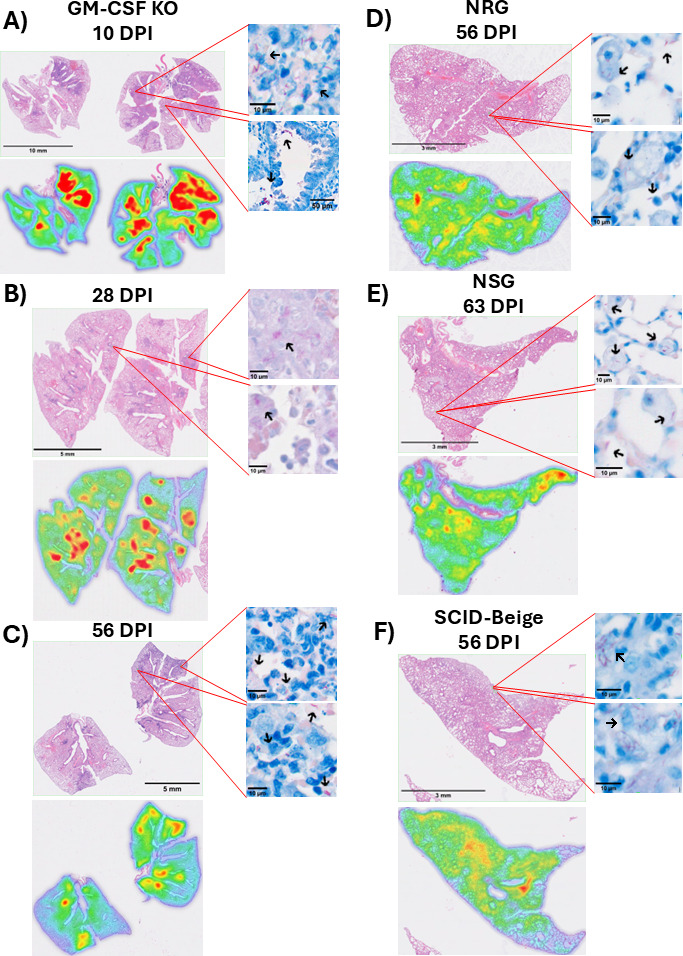
Temporal lung histopathology in GM-CSF KO, NRG, NSG, and SCID-Beige mice. Formalin-fixed paraffin-embedded (FFPE) sections were cut at 5 μm, stained with hematoxylin and eosin (H&E) or Ziehl-Neelsen staining, and imaged at 40×. Each H&E image was converted to heat maps using QuPath software. The heat maps show the lesion areas in red color, green color represents thickening of parenchyma, and blue color uninvolved parenchymal tissue. Inserts to the right of each photo show Ziehl-Neelsen staining for acid-fast positive bacilli (AFB) (fuchsia color pointed by black arrows) in that area. (**A–C**) H&E staining of lung sections and their corresponding heat maps from GM-CSF KO mice at 10 days post-infection (DPI) (**A**), 28 DPI (**B**), and 56 DPI (**C**) or NRG at 56 DPI (**D**), NSG at 63 DPI (**E**), and SCID-Beige at 56 DPI (**F**). In both acute and chronic stages of infection in GM-CSF KO mice (**A–C**), most AFB were found within macrophages, most frequently with foamy appearance (upper insert in **A**, both inserts in **B and C**). Some AFB also appeared extracellularly in the alveoli (lower insert in **A**). In NRG and SCID-Beige mice, macrophage aggregates with intracellular AFB were present in the lung parenchyma; few extracellular bacilli were also evident (inserts in **D and F**, respectively). Pockets of foamy macrophages containing intracellular AFB were seen in alveolar spaces of NSG mice (inserts in **E**).

**Fig 3:**
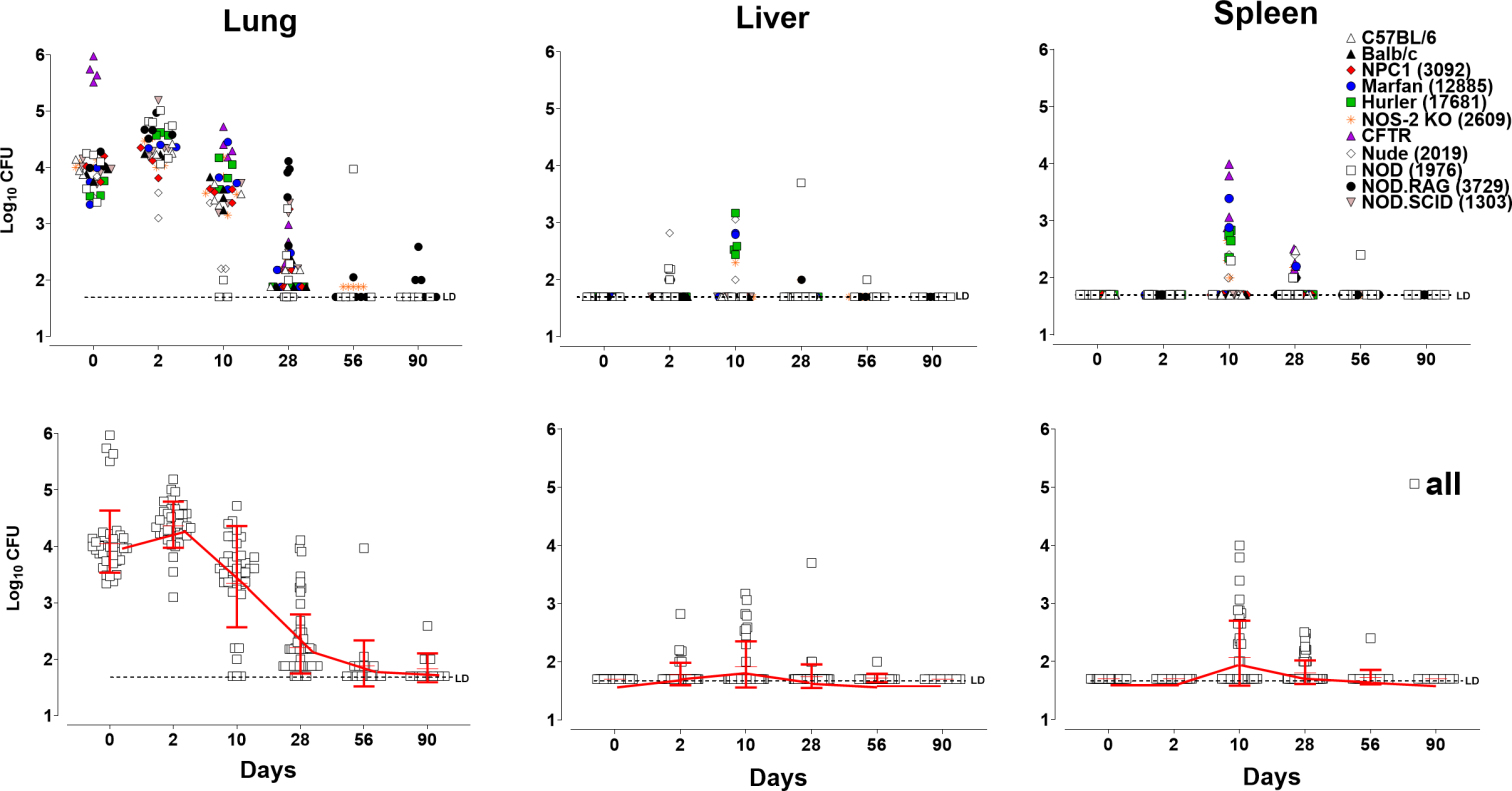
Murine models clearing *M. abscessus* ATCC 19977. Bacterial load (log_10_ CFU; *y*-axis) in C57BL/6, BALB/c, NPC1, Marfan, Hurler, NOS-2KO, CFTR, NUDE, NOD, NOD-RAG, and NOD.SCID mice following aerosol challenge at days 0, 10, 28, 56, and 90 post-infection (*x*-axis). Bacterial loads in the lungs (left graph), livers (center graph), and spleens (right graph) of C57BL/6 (open triangles), BALB/c (blue triangles), NPC1 (red rhomboids), Marfan (blue circles), Hurler (green squares), NOS-2 KO (orange asterisks), CFTR (purple triangles), NUDE (open rhomboids), NOD (open squares), NOD-RAG (black circles), and NOD.SCID (inverted triangles) mice (*n* = 4–5) following aerosol challenge with *M. abscessus* 19977. Upper panel graphs represent individual data points for each mouse strain, and lower panel graphs represent combined data for all mouse strains in the study. For visualization purposes, time on the *x*-axis is not proportional to the number of days between measurements.

We evaluated the establishment and progression of pulmonary, hepatic, and splenic infection with MAB ATCC 19977 smooth morphotype, in diverse strains of immunocompetent and immunodeficient mice. To mimic the most likely route of natural infection in humans, the majority of the studies were performed by infecting mice via the aerosol route using a Glas-Col apparatus or upon intrapulmonary instillation with a microsprayer. When exposing animals via the aerosol route, the lung constitutes the primary site of infection; bacterial seeding of secondary organs like the liver and spleen occurs upon dissemination from the lung. For comparative purposes, we replicated the work of others and infected severe combined immunodeficiency (SCID)-Beige mice only via IV injection (9–11). In this case, all organs are readily infected; however, the liver harbors most of the inoculated bacteria (see below). Models were classified into two groups based on bacterial burden in the lungs after 28 days of infection: (i) non-clearing models, characterized by sustained high bacterial burden >10^3^ colony-forming units (CFU) (Fig. 1 and 2); and (ii) clearing models, defined by either very low or undetectable bacterial burden (Fig. 3).

### Non-clearing MAB murine models

Upon infection via the aerosol route using a Glas-Col apparatus to deliver ~10^4^ CFU of MAB ATCC 19977 smooth morphotype in the lungs of mice, we identified three strains developing chronic (>28 days) pulmonary MAB infection: granulocyte macrophage colony stimulating factor (GM-CSF) KO, NOD.Rag.IL2R gamma chain (NRG), and NOD.SCID.IL2R gamma chain (NSG) mice (Table 1). Non-clearing models were also characterized by either stable (NRG) or increasing (GM-CSF KO and NSG mice) pulmonary MAB burden between days 2 and 10 of infection, as well as productive dissemination to secondary organs including the spleen and liver within a similar timeframe.

**TABLE 1:** Murine strain characteristics and rationale for use in this study

Mouse strain name	Mouse strain ID	Source	Gene mutation	Deficiency	Rationale for use in this study
GM-CSF KO	026812	Breed at CSU/Jackson Laboratory	B6.129S-*Csf2^tm1Mlg^*/J granulocyte-macrophage colony stimulating factor 2 knock-out	Alveolar macrophages maturation	Affects surfactant homeostasis, leading to proteinosis and increased MAB susceptibility
NOD/ShiLtJ	001976	Jackson Laboratory	*NOD/ShiLtJ*, non-obese diabetic	Defects in C5 complement	Used as controls for NSG and NRG mice
NOD/SCID	001303	Jackson Laboratory	*NOD.Cg-Prkdcscid/J*, non-obese diabetic	SCID background lacks functional T and B cells, and NOD background lacks C5 complement	Used as controls for NSG mice
SCID-Beige	250	Charles River	CB17.Cg-*Prkdc^scid^Lyst^bg-J^*/Crl	Lack functional T and B cells (SCID); Lyst mutation affects NK cell granules	Used previously for IV studies.
NU/J Nude	002019	Jackson Laboratory	*Foxn1^nu^*, formerly *Hfh11^nu^*	Abnormal hair growth; athymic: lack T cells, partial defect in B cells, and lack cell-mediated immunity	Evaluate role of T cells against MAB
NSG	005557	Jackson Laboratory	NOD.Cg-*Prkdc^scid^ Il2rg^tm1Wjl^*/SzJ NOD *scid* gamma	Absence of T and B cells (SCID); missing IL-2R gamma chain leads to defective IL-2, IL-4, IL-7, IL-9, IL-15, IL-21 signaling. Lack functional NK cells because of impaired IL-15	Severely immunodeficient
NRG	007799	Jackson Laboratory	NOD.Cg-*Rag1^tm1Mom^ Il2rg^tm1Wjl^*/SzJ	Absence of T and B cells (Rag1); missing IL-2R gamma chain leads to defective IL-2, IL-4, IL-7, IL-9, IL-15, IL-21 signaling. Lack functional NK cells because of impaired IL-15	Severely immunodeficient
BALB/cJ	000651	Jackson Laboratory		Fully immunocompetent	Used as control for NPC1 mice
C57BL/6J	000664	Jackson Laboratory		Fully immunocompetent	Used as control for several strains
NOS-2 KO	002609	Jackson Laboratory	B6.129P2-*Nos2^tm1Lau^*/J NOS-2 KO	Lacking NO production leading to high TB susceptibility	Lower NO production reported in CF patients
Marfan	012885	Jackson Laboratory	B6.129-*Fbn1^tm1Hcd^*/J	Mutation in fibrillin-1, develop manifestations of Marfan syndrome: proximal aortic aneurysms, mitral valve thickenings, pulmonary alveolar septation defects, mild thoracic kyphosis, and skeletal myopathy	Share phenotypic features reported in patients with pulmonary MAB infection
Hurler	017681	Jackson Laboratory	B6.129S-*Idua^tm1.1Kmke^*/J	Lysosomal storage disease (Hurler syndrome)	Lysosomal dysfunction
NPC1	003092	Jackson Laboratory	BALB/cNctr-*Npc1^m1N^*/J Niemann Pick Type C1	Accumulation of cholesterol and fatty acids due to defects in lysosomal export	Lysosomal dysfunction
NOD/Rag	003729	Jackson Laboratory	NOD.129S7(B6)-Rag1tm1Mom/J	Rag background lacks functional T and B cells, and NOD background lacks C5 complement	Used as controls for NRG mice
CFTR	FABP-CFTR	Animal Resource Center at Case Western University	Cftrtm1KthTg(FABPCFTR)1Jaw /Cwr	F508del mutation in Cftr; expresses human Cftr from the fatty acid binding protein promoter (FABP)	Cftr expressed in intestines infrequent intestinal obstruction and labeled “gut-corrected.” However, human CFTR may be expressed elsewhere in the mouse

In GM-CSF KO mice, a 10,000-fold increase in pulmonary bacteria load was observed within the first 10 days post-challenge. Thereafter, the number of CFUs declined rapidly until day 28 post-challenge and plateaued during days 28 to 56 post-challenge (Fig. 1A). The trend for CFUs recovered from livers and spleens of GM-CSF KO mice was similar to that seen in the lungs. Specifically, bacterial numbers peaked in both organs at day 10 (~10^4^ CFU), declining thereafter to almost reach the limits of detection (LD) of the assay by day 28.

In contrast to the course of MAB infection observed in GM-CSF KO mice (exponential growth followed by a rapid decline), the pulmonary MAB burden in NRG and NSG mice slowly but steadily increased between days 0 and 10 post-challenge, remaining at 10^4^ to 10^5^ until day 90 (the last time point tested). It is important to note that animal intervariability in the NRG group was lower than that of the NSG mice, i.e., at day 90, NRG and NSG log_10_ lung CFUs of 4.75 ± 0.3 and 4.48 ± 1.6, respectively. CFUs in livers and spleens of NRG and NSG mice also increased steadily until day 90, when they reached ~10^4^ and 10^3^, respectively.

We also reevaluated the course of infection in SCID-Beige mice (strain #250; Charles River), lacking T, B, and NK cell activity, following IV injection with ~10^6^ CFUs of MAB ATCC 19977 (Fig. 1B). As reported by us (9) and others (10, 11), in this commonly used model of MAB infection, approximately 90% of the injected bacilli were implanted in the liver, whereas 10% was found in the spleen and only 1% was located in the lungs. Bacterial burden fluctuated slightly throughout the course of infection; however, it never deviated much from the initial seeding of ~10^3^, 10^4^, and 10^5^ CFUs in the lung, spleen, and liver, respectively. It is important to note that some mice developed symptoms of vertigo following IV infection, likely due to brain or inner ear involvement, which necessitated immediate euthanasia.

Histopathology was evaluated in lung sections from GM-CSF KO (Fig. 2A through C), NRG (Fig. 2D), NSG (Fig. 2E), and SCID-Beige (Fig. 2F) mice at specific time points during acute (GM-CSF KO mice) and chronic (GM-CSF KO, NSG, NRG, and SCID-Beige mice) stages of infection. To monitor the inflammatory response and presence of acid-fast positive bacilli (AFB), lung sections were processed by hematoxylin and eosin (H&E) or Ziehl-Neelsen staining, respectively. The GM-CSF KO model showed inflammation as early as 2 days post-infection (DPI) (data not shown), and over the course of infection, the highest levels of inflammation were seen at 10 DPI (Fig. 3A). Regions of inflammation were characterized by thickening of the parenchyma (green on heat maps), including small bronchioles and effaced alveolar architecture with cellular aggregations forming granulomas (red on heat maps). The granulomas consisted of macrophages, some with abundant cytoplasmic foamy vacuolation (also referred to as “foamy” macrophages), abundance of neutrophils, and some lymphocytes. By day 28 post-infection (Fig. 2B and C), the inflammation subsided, and the size of each granuloma was typically smaller when compared to 10 DPI (heat maps in Fig. 2A through C). This pattern persisted through 56 DPI, with granulomas consisting of large concentrations of macrophages (many as foamy), a reduced number of neutrophils, and higher infiltration of lymphocytes. AFB (inserts in Fig. 2A through C with black arrows pointing to AFB) were found in all lung samples collected from GM-CSF KO mice, especially during acute stages of infection (10 DPI; Fig. 2A). In both acute and chronic stages of infection, most AFB were observed within macrophages, predominantly with foamy appearance (upper insert in Fig. 2A and both inserts in Fig. 2B and C). However, some AFB appeared extracellularly in the alveoli at all time points (lower insert in Fig. 2A, black arrows). Lung inflammation in NRG, NSG, and SCID-Beige mice (Fig. 2D through F, respectively) presented differently from GM-CSF KO mice. Specifically, inflammation was only evident during the chronic state of infection (56–63 DPI) and presented as extensive thickening of the parenchyma. As expected, based on their background, lymphocyte infiltration was absent in these mice. Interestingly, pockets of macrophage accumulation (many as foamy) were present in NSG mice. Most AFB were localized within macrophages, with only a few found extracellularly (inserts in Fig. 2E).

From these studies, we concluded that GM-CSF KO, NRG, NSG, and SCID-Beige mice sustain MAB infection chronically. In GM-CSF KO mice, chronic infection and pathology are preceded by high bacterial burden that wanes after 10 days of infection and thereafter remains steady for at least 56 days, whereas in NSG and NRG mice, chronic infection is characterized by a gradual—yet subdued—increment in bacteria load, accompanied by mild inflammation.

### Clearing MAB murine models

Figure 3 shows the course of MAB ATCC 19977 infection in the lungs, spleens, and livers of multiple mouse strains (described in Table 1), including immunocompetent mice (BALB/c and C57BL/6), mice lacking function in the cystic fibrosis transmembrane conductance regulator (CFTR) or nitric oxide synthase 2 (NOS-2-KO), mice developing lysosomal storage disease (NPC1 and Hurler) or connective tissue disorders (Marfan1), diabetes-prone mice lacking C5 of the complement system (NOD), or mice lacking T and B cells in the NOD background (NOD-RAG and NOD-SCID). The rationale to test these mouse strains was based on reports documenting reduced nitric oxide concentration in the airways of CF patients (12, 13), phenotypic similarities between patients with Marfan syndrome and those developing non-tuberculous mycobacterium (NTM) infections (14, 15), and manipulation of lysosomal function by pathogenic mycobacteria, particularly NPC1 (16), respectively. Except for CFTR mice infected via intrapulmonary instillation with 10^6^ CFU, all other strains of mice were challenged via the aerosol route using the Glas-Col apparatus (Fig. 3), as described for non-clearing models above in Fig. 1. Interestingly, despite the broad range of alterations impacting different arms of the immune system in these murine strains, the course of MAB infection in all of them was remarkably similar. There was an average of 0.5 log_10_-fold increase (ns) in the pulmonary bacterial load in all mouse strains at day 2 post-challenge, except for CFTR mice in which CFUs decreased at this time point. In contrast to non-clearing models described above, CFUs in clearing models declined rapidly between days 2 and 10 post-infection, and this trend continued until day 28 post-challenge. Finally, CFU numbers in most mice reached the LD between day 56 and day 90 post-challenge. Bacteria were transiently recovered from the liver and spleen of some mice at days 2, 10, and 28; however, the overall trend showed minimal extrapulmonary dissemination and/or colonization. Therefore, we concluded that these mouse strains are generally effective at clearing MAB and are unable to develop progressive or sustained infection.

### Comparison of intrapulmonary and Glas-Col inhalation aerosol methods

**Fig 4:**
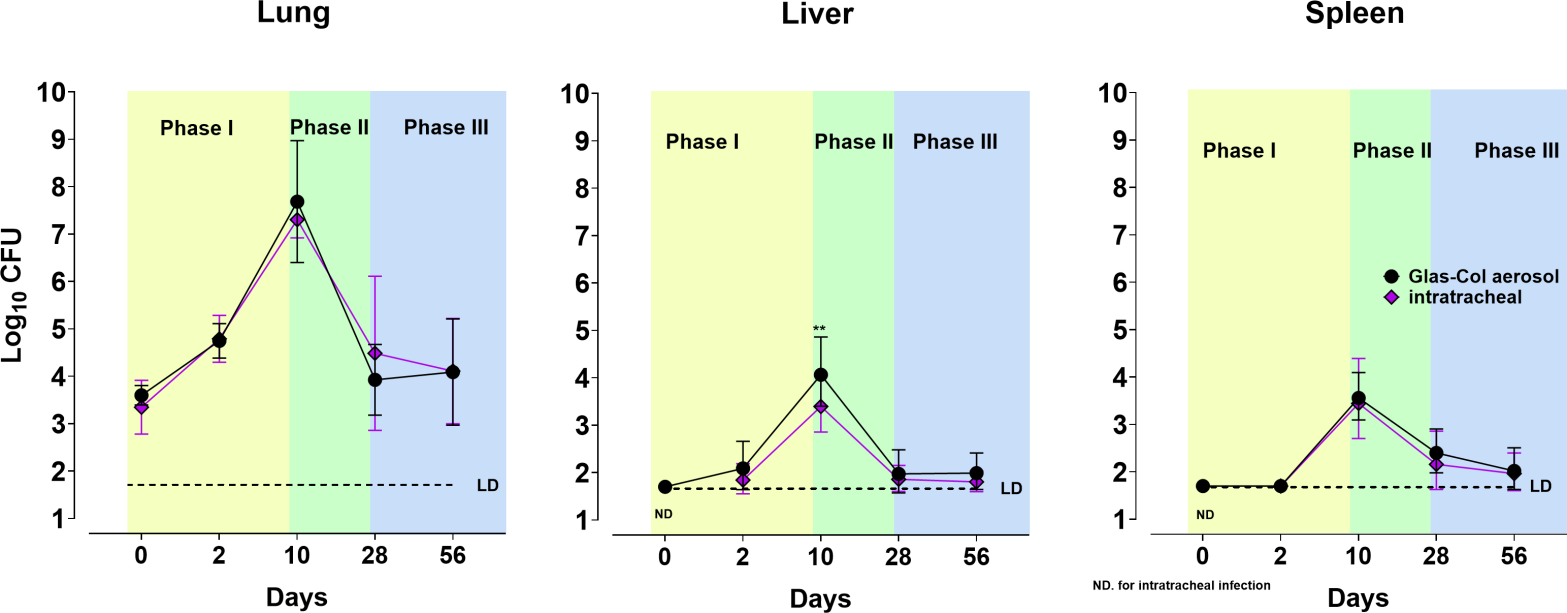
Comparable infection time course in GM-CSF KO mice exposed to *M. abscessus* via intrapulmonary aerosol vs Glas-Col apparatus. Bacterial load in GM-CSF KO mice following aerosol challenge via intrapulmonary aerosol (purple rhomboids) or Glas-Col inhalation aerosol (black circles). Bacterial loads (*y*-axis: log_10_ CFU) in the lungs (left graph), livers (center graph), and spleens (right graph). Bacterial loads were measured at days 0, 2, 10, 28, 56, and 90 post-infection (*x*-axis: days). Colored areas in graphs show three distinctive phases in the course of infection: yellow = phase I; green = phase II; and blue = phase III. Combined data from more than 10 experiments performed with intrapulmonary aerosol and 3 experiments performed with Glas-Col apparatus. Data were analyzed using an unpaired t-test. For visualization purposes, time on the *x*-axis is not proportional to the number of days between measurements.

Given that laboratories may choose to perform mouse aerosol infections using either the Glas-Col apparatus or a microsprayer device for intrapulmonary instillation, we compared the effectiveness of both techniques in GM-CSF KO mice (Fig. 4). Interestingly, at multiple time points (0, 10, 28, 56, and 90 DPI), there were no significant differences in CFUs between both methods (except in the livers at day 10 post-challenge). Overall, both methods resulted in a similar course of infection and sustained live MAB equal to or higher than 10^3^ CFUs in the lungs at all time points. We concluded that in the GM-CSF KO inhalation aerosol model, the course of MAB infection can be divided into three distinct stages (Fig. 4): phase I, an acute phase from day 0 to day 10 post-infection, marked by a logarithmic increase in CFU numbers; phase II, an early chronic stage from day 10 to day 28, characterized by a significant decrease in CFUs, reaching ~10^4^ CFU; and phase III, a chronic state established between day 28 and day 90 post-infection, with a steady bacterial load of ~10^3^ CFUs in the lungs.

### Validation of NRG, GM-CSF KO, and SCID-Beige mouse as MAB models for drug screening

We also assessed the potential of the murine MAB models described above to test the antimicrobial efficacy of several antibiotics commonly used in clinical treatment. Mice were treated with monotherapy both in acute—aerosol- or IV-infected GM-CSF KO and SCID-Beige mice, respectively—or chronic regimens—aerosol-infected NRG and GM-CSF KO mice, or IV-infected SCID-Beige. Drug efficacy at the end of the study was determined by the statistically significant reduction in CFU numbers in lungs, spleens, or livers of treated mice relative to CFU counts in similar samples from untreated controls. In acute regimens, antibiotic treatment started on day 2 post-infection and continued for 9 days. In chronic regimens, antibiotic treatment was initiated on day 28 or 56 post-infection and administered for 4 consecutive weeks.

**Fig 5:**
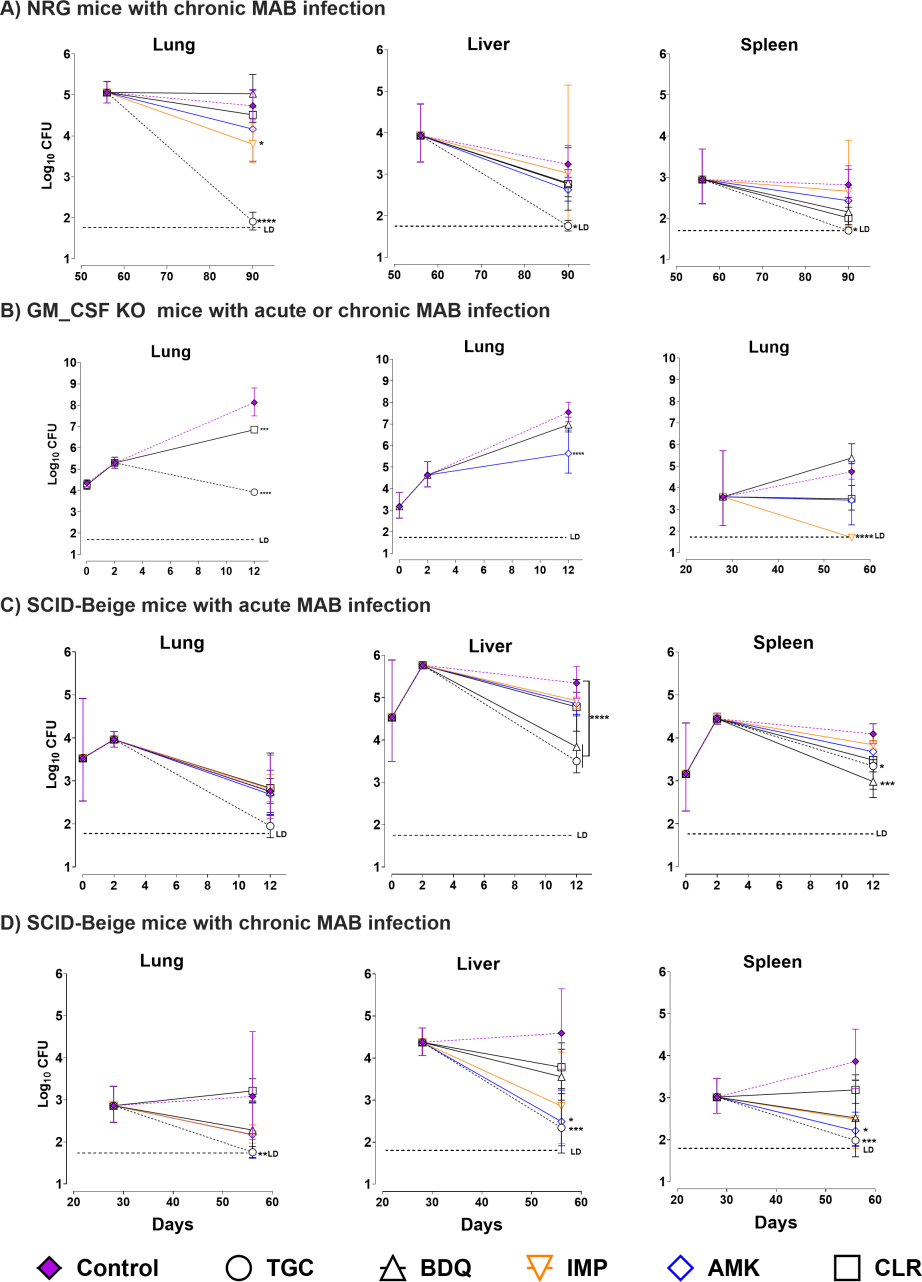
Antimicrobial screening in murine models of *M. abscessus* infection. The bacterial load was measured as log_10_ CFU in three mouse models: NRG, GM-CSF KO, and SCID-Beige after antibiotic treatment (*n* = 5). (**A**) Bacterial loads were determined in the lungs (left), liver (center), and spleen (right) of NRG mice. Treatments began on day 56 post-infection and continued for 4 weeks. (**B**) Bacterial load in the lungs of GM-CSF KO mice during acute (left and center) and chronic (right) infections. For acute infection, treatment started on day 2 post-infection and lasted 10 days, while chronic infection treatment started on day 28 post-infection and continued for 4 weeks. (**C**) Bacterial load in the lungs (left), livers (center), and spleens (right) of SCID-Beige mice, with treatment beginning on day 2 post-infection for a duration of 10 days. (**D**) Bacterial load in the lungs (left), livers (center), and spleens (right) of SCID-Beige mice, with treatment beginning on day 28 post-infection for a duration of four weeks. The mice were either untreated (purple rhomboids) or treated with monotherapy regimens of TGC at 50 mg/kg (black circles); BDQ at 25 mg/kg (black triangles); IMP at 100 mg/kg (orange triangles); AMK at 150 mg/kg (blue rhomboids); or CLR at 250 mg/kg (open squares). Drugs were administered 5 or 7 days a week with oral gavage for BDQ and CLR, and subcutaneous injection for AMK, IMP, and TGC. The dosing frequency was once daily for all drugs except IMP and TGC, which were administered twice daily. Data are presented as mean log_10_ CFU with standard deviation. Differences between groups were analyzed by one-way analysis of variance with Dunnett’s test.

Despite some variability in antibiotic efficacy across the different models—acute vs chronic, IV vs aerosol, mouse strain, and organ—consistent trends emerged. TGC was highly effective as monotherapy in both acute and chronic models and, in multiple instances, reduced bacterial levels to the LD (Fig. 5). Specifically, TGC treatment in chronically infected NRG mice resulted in 2.8, 1.5, and 1.1 log_10_ CFU reduction in the lungs (*P* < 0.0001), liver (*P* < 0.01), and spleen (*P* < 0.01), respectively (Fig. 5A). As we recently published in the acute GM-CSF KO model (17), TGC treatment dramatically reduced bacterial burden by 4.5 log_10_ CFU (*P* < 0.0001) (Fig. 5B). Likewise, TGC treatment was also effective in SCID-Beige mice acutely or chronically infected with MAB (Fig. 5C and D). IMP was also very effective, reducing the lung CFUs by 0.9 log_10_ (*P* < 0.01) and 3.1 log_10_ CFU (*P* < 0.0001) in chronically infected NRG and GM-CSF KO mice, respectively (Fig. 5A and B). Interestingly, antibiotics like CLR and AMK commonly used in the clinic to treat MAB-infected patients were only effective in the lungs of acutely infected GM-CSF KO mice, leading to a 1.1 log_10_ CFU (*P* < 0.0001) and 1.8 log_10_ CFU (*P* < 0.0001) reduction, respectively (Fig. 5B, center graph); however, CLR and AMK were ineffective in the lungs of the three chronic models (Fig. 5A, B, and D). Finally, BDQ was mostly ineffective except in the liver and spleen of acutely, IV-infected SCID-Beige mice. Statistically significant differences after antibiotic therapy of IV-infected SCID-Beige mice were more likely to be detected in livers and spleens rather than lungs, with CFU variability having a greater impact on the narrower dynamic range for bacterial pulmonary burden.

## DISCUSSION

Patients with pulmonary MAB infection develop long-term chronic infections, requiring prolonged treatment with multiple oral and parenteral antibiotics over 12–24 months. However, these regimens are often associated with poor bactericidal activity and significant toxicity (2, 3). There is a dire need for new drugs and regimens to treat MAB infections, with the evaluation of these therapies hinging on the availability of well-characterized small animal models. In pursuit of finding a suitable model to test drug efficacy against MAB, we evaluated the progression of acute (<28 days) and chronic MAB infection (>28 days) in the lungs, liver, and spleen of 15 mouse strains, each with diverse genetic mutations leading to immunological, lysosomal, and connective tissue disorders or their respective controls. Models were classified as non-clearing (Fig. 1 and 2) or clearing (Fig. 3), depending on their ability to sustain or control high pulmonary burden of >10^3^ CFU at 28 days post-challenge. Furthermore, those developing chronic pulmonary infection (non-clearing) also had sustained or increasing pulmonary MAB burden early during infection (days 2 to 10), as well as productive dissemination to liver and spleen. Taking into consideration the LD of the assay used here (1.6–1.88 log_10_ CFU), we selected the cutoff at 10^3^ CFUs to enable detection of at least a 10-fold reduction in bacterial load upon drug treatment. Four mouse strains with progressive infection were identified: two new mouse strains (NSG and NRG), in addition to the previously reported GM-CSF KO (18) and IV-infected SCID-Beige mice (9), and their potential use for preclinical drug testing was evaluated.

Murine strains infected herein were specifically selected based on our current understanding of clinical conditions that predispose individuals to NTM disease, as well as potential underlying pathophysiological mechanism(s) involved. Interestingly, the course of MAB infection was similar between control mice and the majority of the tested murine strains, with MAB burden declining to the LD by day 28 post-infection. Specifically, CFTR KO mice cleared MAB infection indistinctly from C57BL/6 background controls. However, CFTR KO mice do not develop the progressive CF-like lung pathology observed in humans (19, 20). Because pulmonary MAB infections often occur after lung structural/functional damage caused by a chronic state of inflammation and scarring (e.g., CF, bronchiectasis, previous TB history, etc.), we suggest it is not a systemic immunodeficiency (as in SCID-Beige and NSG/NRG mice) but the local pulmonary alteration which creates a suitable environment for establishment and progression of MAB infection. We had previously reported that βENaC mice, a second mouse model of CF with mucous hyperproduction, also cleared MAB (21). Likewise, NOS-2 KO, Marfan, Hurler, and NPC1 mice displayed similar courses of MAB infection to the control mice. While reduced nitric oxide production, anatomic alterations, and lysosomal dysfunction might contribute to MAB colonization and disease progression in humans, MAB pathogenesis is probably multifactorial and difficult to model in animals with monogenetic deficiencies causing these conditions, respectively. Although we did not evaluate other clinical isolates of MAB, rough morphotypes, or alternative routes of infection in these mouse strains, this could represent yet another example of the well-known limitations in translating findings from humans to mice (22–24). Alternatively, it highlights our limited understanding of immune and non-immune factors (hormonal, metabolic, structural, etc.) conducive to NTM control vs susceptibility, and how to effectively model these in animals.

Three out of four murine strains developing chronic NTM infection—SCID-Beige, NRG, and NSG—are highly immunocompromised, lacking T, B, and NK cells. Interestingly, simultaneous deficiency of these three cell types was required to maintain chronic MAB infection in these murine strains. In fact, mice with T cell deficiency (NUDE), or deficient for both B and T cells (NOD.SCID and NOD.Rag), cleared the infection similarly to immunocompetent mice, suggesting redundant immunological mechanisms may counteract MAB. In contrast to NOD.SCID and NOD.Rag mice, NSG and NRG mice also lack the IL-2R gamma chain shared by several cytokine receptors including IL-2, IL-4, IL-7, IL-9, IL-15, and IL-21. Thus, its deficiency is expected to have pleiotropic effects including regulation of cell proliferation, survival, cytolytic activity, and differentiation (25), hampering identification of specific mechanism(s) leading to MAB survival. Despite the potential use of SCID-Beige and NSG/NRG strains in testing of anti-MAB drugs, we argue these models do not recapitulate human disease caused by MAB infection, as patients do not have such extensive levels of systemic immunodeficiencies, further exemplified by the minimal lung pathology in these mouse strains during MAB chronic infection.

GM-CSF KO mice represent the fourth model of non-clearing MAB infection described herein. We had previously reported similar findings using this mouse strain (18); however, the source of these mice has changed since our initial report, and confirmatory studies were warranted. The GM-CSF KO model is highly reproducible with the course of infection having three distinctive phases: phase I/acute defined by a logarithmic CFU increase between days 0 and 10 post-challenge; phase II/early chronic consisting of a rapid CFU reduction between days 10 and 28 post-infection; and phase III/chronic, characterized by a sustained CFU level between days 28 to 90 post-infection.

A major motivation to perform these studies was to identify animal models of MAB infection for drug validation. Results suggest that antibiotics such as TGC and IMP are effective in both acute and chronic models tested here. Conversely, antibiotics like AMK and CLR currently used in the clinics to treat MAB-infected patients primarily show efficacy in acute models. Finally, BDQ had minimal activity against acute or chronic MAB infections. It remains to be determined whether antibiotic activity here is related to different bacterial physiological states in acute vs chronic models (i.e., actively dividing vs non-replicating), altered antibiotic pharmacokinetic (PK)/pharmacodynamic (PD) profiles due to tissue pathology, or the multiple antimicrobial resistance mechanisms present in MAB, including *erm(41*) gene-mediated macrolide resistance, drug efflux pumps, etc. (1). Despite its strong antimicrobial activity, TGC has a boxed warning associating increased mortality in those patients taking this medication (https://www.pfizermedicalinformation.com/tygacil/boxed-warning). Furthermore, TGC has poor tolerance, especially with parenteral delivery. Efforts are being made to explore an inhalation route to improve tolerability (26, 27). Based on producing comparable TGC plasma exposure quantified as *f*AUC(0–24), the mouse equivalent dose of TGC compared to the approved human dosing of 50 mg q12h was calculated as 1.66 mg/kg q12h (28), substantially lower than 50 mg/kg q12h used herein. The primary purpose of the studies described in our manuscript was to compare new murine models for screening antibacterials against MAB infections, not to evaluate and characterize which TGC dose would be minimally effective to control pulmonary MAB infection. Thus, we decided to use a TGC dosing regimen that would be well tolerated in the different mouse strains but would surely not be limited in its efficacy (if there were any) by a too low systemic exposure. Thus, this is a proof of concept of the model and its ability to detect antibacterial activity against MAB infection, rather than an assessment of the minimally effective TGC dose and its corresponding exposure necessary to control these infections.

We have not yet evaluated combination therapy in these models of MAB infection but intend to do so soon to discover drug combinations that minimize toxicity and resistance, while improving bacterial eradication.

We prefer the aerosol route of infection as it most closely mimics the route of infection in humans; however, many laboratories are not equipped with a Glas-Col apparatus or have access to microsprayer. When infection is performed via the IV route, bacterial seeding preferentially occurs in the liver, a key organ catabolizing and/or excreting antibiotic. It remains to be determined if and how PK/PD parameters could be affected upon hepatic infection and the implications in drug studies. Furthermore, we and others (29) have noticed that some mice develop vertigo upon IV infection and brain or inner ear involvement, and for these reasons, we discourage this route of infection.

We acknowledge that our studies have several limitations. Infections were only performed with the smooth morphotype of MAB ATCC 19977, as this strain is widely used in the field, its genome has been annotated, and it belongs to the dominant circulating clone I family which has been found to be overrepresented in NTM-infected CF and non-CF patients (30). A different result might be obtained with other MAB isolates taking into consideration strain heterogeneity. Moreover, as mentioned above, use of animal models for drug testing depends on the quick availability of large quantities of age-matched animals needed to perform these studies. Unfortunately, commercial availability of GM-CSF KO, NSG, and NRG is limited by their high cost and lack of rapid turnaround by the supplier. If these animals were to be used by several research groups on a larger scale, these problems need to be addressed by the funding and research communities.

## MATERIAL AND METHODS

### Bacterial inoculum preparation

MAB ATCC 19977, smooth morphotype was obtained from the American Type Culture Collection (Manassas, VA), reconstituted, and cultured following established protocols for the creation of frozen stocks. One milliliter of MAB stock was added to 5 mL of 7H9 broth supplemented with 10% oleic acid, albumin, dextrose, catalase (OADC), 0.5% glycerol, and 0.05% Tween 80. The cultures were stirred for 24 h to achieve exponential growth phase and harvested at OD_600_ = 0.62, equivalent to ~1 × 10^8^ CFU/mL for experimental use.

### Animals

The mouse strains, nomenclature ID along with their corresponding gene mutation, and derived immune phenotype used in this study are summarized in Table 1.

### Mouse infection procedure

Three methods were used to infect mice:

#### Intrapulmonary aerosol

Inoculum was prepared with a 1,000-fold dilution of a fresh culture (OD _600_ = 0.62) in sterile 0.9% endotoxin-free saline, (~1 × 10^5^ CFU/mL). Two doses of 50 µL of inoculum suspension were delivered intratracheally to each animal as an intrapulmonary spray instillation using a high-pressure syringe device (PennCentury), for a targeted dose of ~10^4^ CFU/mouse (21, 26).

#### Glas-Col chamber aerosol

Animals were infected with a high-dose aerosol infection of MAB ATCC 19977 using an inhalation exposure system (Glas-Col, Terre Haute, IN) calibrated to deliver ~10^4^ CFU to the lungs.

#### IV infection

Mice were warmed under a heat lamp to allow for vasodilation, secured in a restraint, and infected via IV injection of the lateral vein with 0.1 mL of ~10^7^ CFU/mL bacteria to deliver ~10^6^ CFU/mouse (9).

For each of the three routes of infection, bacterial deposition in the lungs was confirmed by humanely euthanizing mice (*n* = 3–5) via CO_2_ narcosis 2–3 h after infection, and their lungs were harvested for bacterial quantification as described below. During infection, mice were monitored daily for clinical observations (e.g., inactivity, rough fur, hunched posture, increased respiratory rate, or effort), and their body weights were taken weekly.

### Bacterial burden enumeration

Lung, liver, and spleen were harvested for bacterial burden enumeration at defined time points post-challenge. Tissues were homogenized using the Precellys Tissue Homogenizer (Precellys Lysing Kit, 220325-830). Subsequently, 100 µL of a seven-point serial fivefold dilution of each homogenate was plated on Middlebrook ^7^H11 agar (Millipore, M0428-500G) supplemented with carbenicillin (Sigma-Aldrich, C1389-1G) and cycloheximide (GoldBio, C-930-10). Plates were incubated for 3–5 days at 37°C until visible CFU could be enumerated.

### Antimicrobials

CLR (TCI, Cat# C2220), AMK disulfate (Thermo Scientific Chemical, cat #J63862.14), BDQ fumarate (LKT Labs, cat# B165121), TGC (ChemShuttle cat# 101150), and IMP (Avachem Scientific, Cat# 1720) were purchased. CLR was administered at 250 mg/kg and prepared with 0.5% carboxymethylcellulose sodium salt (Sigma-Aldrich Cat# C4888 S00G) containing 0.5% Tween 80. AMK was dosed at 150 mg/kg and dissolved in 0.9% saline. BDQ was administered at 25 mg/kg and prepared by dissolving in an acidified 20% (wt/vol) 2-hydroxypropyl-β-cyclodextrin (Sigma, Cat#332593) solution, and the final pH was adjusted to 3.5. TGC was administered at 50 mg/kg twice daily and dissolved immediately before administration in 0.9% saline. IMP was administered at 100 mg/kg and prepared in water.

In the acute model of infection (GM-CSF KO and SCID-Beige), drug treatment started on day 2. In chronic models of infection, drug treatment started on day 28 or 56 for GM-CSF and NRG mice, respectively. Drugs were administered once (CLR, AMK, BDQ) or twice (TGC, IMP) daily by oral gavage (CLR, BDQ) or subcutaneous administration (AMK, TGC, IMP) for the indicated times.

### Histopathology and lesion scoring

The lungs were fixed in 4% paraformaldehyde for 48 h and then embedded in paraffin for histopathology. Sections were cut at 5 µm, stained with H&E, and scanned at 40× magnification using multispectral automated PhenoImager (Akoya Biosciences) for histopathological evaluation. The extent of lung lesion burden was quantified in blinded digital images using an open-source QuPath software for image analysis as described previously (31).

### Statistical analysis

Bacterial burden data were expressed as CFU, log_10_-transformed, and analyzed using GraphPad Prism version 9.5.1 (GraphPad software, La Jolla, CA). Statistical analysis was performed using unpaired *t*-test or one-way analysis of variance and Tukey’s or Dunnett *post hoc* tests. Significance was defined as: *P < 0.05, **P < 0.01, ***P < 0.001, ****P < 0.0001.
